# A Novel Computational Approach for Identifying Essential Proteins From Multiplex Biological Networks

**DOI:** 10.3389/fgene.2020.00343

**Published:** 2020-04-21

**Authors:** Bihai Zhao, Sai Hu, Xiner Liu, Huijun Xiong, Xiao Han, Zhihong Zhang, Xueyong Li, Lei Wang

**Affiliations:** ^1^College of Computer Engineering and Applied Mathematics, Changsha University, Changsha, China; ^2^Hunan Provincial Key Laboratory of Industrial Internet Technology and Security, Changsha University, Changsha, China; ^3^Hunan Provincial Key Laboratory of Nutrition and Quality Control of Aquatic Animals, Changsha University, Changsha, China

**Keywords:** identification of essential proteins, protein interaction network, tensor, multiplex biological networks, random walk, Markov chain, gene expression, yeast

## Abstract

The identification of essential proteins can help in understanding the minimum requirements for cell survival and development. Ever-increasing amounts of high-throughput data provide us with opportunities to detect essential proteins from protein interaction networks (PINs). Existing network-based approaches are limited by the poor quality of the underlying PIN data, which exhibits high rates of false positive and false negative results. To overcome this problem, researchers have focused on the prediction of essential proteins by combining PINs with other biological data, which has led to the emergence of various interactions between proteins. It remains challenging, however, to use aggregated multiplex interactions within a single analysis framework to identify essential proteins. In this study, we created a multiplex biological network (MON) by initially integrating PINs, protein domains, and gene expression profiles. Next, we proposed a new approach to discover essential proteins by extending the random walk with restart algorithm to the tensor, which provides a data model representation of the MON. In contrast to existing approaches, the proposed MON approach considers for the importance of nodes and the different types of interactions between proteins during the iteration. MON was implemented to identify essential proteins within two yeast PINs. Our comprehensive experimental results demonstrated that MON outperformed 11 other state-of-the-art approaches in terms of precision-recall curve, jackknife curve, and other criteria.

## Introduction

Essential proteins are necessary for the survival of living organisms. The identification of essential proteins can help us to understand the basic requirements of living organisms, and it can also play an important role in drug design (Dubach et al., 2017), genetic disease diagnosis (Zeng et al., 2017), and drug synergy prediction in cancers (Li et al., 2018). Traditional experimental approaches, such as gene knockouts (Narasimhan et al., 2016), RNA interference (Inouye, 2016), and Knockout Sudoku (Baym et al., 2016), are time-consuming and costly. Over the last few decades, high-throughput technologies have produced a tremendous amount of protein interaction network (PIN) data that provide us with new opportunities to detect essential proteins through the use of computational approaches. A number of network topology-based centrality approaches have been proposed to predict essential proteins, and these approaches include Degree Centrality (DC) (Hahn and Kern, 2004), Information Centrality (IC) (Stephenson and Zelen, 1989), Closeness Centrality (CC) (Wuchty and Stadler, 2003), Betweenness Centrality (BC) (Joy et al., 2005), Subgraph Centrality (SC) (Estrada and Rodriguez-Velazquez, 2005), and Neighbor Centrality (NC) (Wang et al., 2011).

Unfortunately, these approaches are often plagued by noise and errors, which can result in biases and low confidence in protein–protein interaction (PPI) networks. To provide accurate prediction results, the integration of different types of biological data has become an important and popular strategy. A number of approaches have been developed to facilitate the prediction of essential proteins by combining PINs with multisource biological data. For example, Gene Ontology (GO) annotations were used as a bioinformatics tool to predict essential proteins in several single-cell PINs, such as those from *Escherichia coli, Saccharomyces cerevisiae*, and *Drosophila melanogaster* (Hsing et al., 2008). A prediction model called integrating orthology with PPI network (ION) (Peng et al., 2012) was proposed to infer essential proteins by integrating orthologous information and the topological characteristics of PINs. In the United complex Centrality (UC) (Li et al., 2015) method, protein complexes were also combined with the topological features of PINs to detect essential genes. After analyzing the correlations between domain characteristics and essential proteins, Peng et al. (2015) designed an approach named unite domain and network centrality (UDoNC) for the prediction of essential proteins in yeast PINs. Li et al. (2012) and Zhang et al. (2013) developed two types of prediction models called prediction of essential proteins centrality (PeC) and co-expression weighted by clustering coefficient method (CoEWC) to infer essential proteins by fusing gene expressions and topological characteristics of PINs, respectively. In our previous studies, we proposed a prediction method called predictive model based on overlapping essential modules (POEM) (Zhao et al., 2014) to measure the essentiality of proteins by detecting overlapping essential modules based on the modularity of essential proteins. Lei et al. (2018) designed a method called AFSO_EP for the prediction of essential proteins based on the artificial fish-swarm algorithm. In this method, the network topology, gene expression, GO annotation, and subcellular localization information were utilized. Zhang W. et al. (2019) proposed a new method to discover essential proteins, named predicting essential proteins by integrating network topology, expression profile, GO annotation and subcellular localization (TEGS), based on integrating network topology, gene expression profiles, GO annotation information, and protein subcellular localization information. In the fusing the dynamic PPI networks (FDP) approach Zhang F. et al. (2019), active PINs were constructed first and then they were fused into a final network according to the networks' similarities. Finally, a new approach for identification of essential proteins was proposed by considering orthologous property and topological properties in the network.

A common characteristic and limitation of these approaches, however, is that they complete the prediction of essential proteins using only a single network of relationships between proteins. Currently, PINs are not the only large-scale network datasets, as protein–DNA interactions and signaling-regulatory pathway interaction data are also stored in dedicated databases (Valdeolivas et al., 2019). Additionally, other interactions such as the co-expression network established from gene expression profiles and the co-annotation network constructed from GO annotations can be derived. Each interaction data source has its own meaning or relevance and can play a different role in the prediction of essential proteins. These approaches mentioned above classically aggregated multiple interaction networks into a single and unique network, which tends to dismiss the topologies and features of the individual interaction networks. The convention of representing different types of interactions in a system with a single type of link is no longer a panacea for network science (De Domenico et al., 2015). The multiplex network offers us an alternative, in that it is a collection of networks sharing the same nodes; however, the edges belong to different categories or represent interactions of different natures (Didier et al., 2015). More recently, various applied studies have been adapted to multiplex networks. Valdeolivas et al. (2019) extended the Random walk algorithm to multiplex networks by building an *nL* × *nL* heterogeneous matrix in which *n* and *L* represent the number of nodes and layers of the multiplex network, respectively. Wang et al. (2018) compressed the multiple networks into two feature matrices and performed conserved functional modules detection by multi-view non-negative matrix factorization. In a newly proposed link prediction algorithm (Samei and Jalili, 2019) for multiplex networks, both intra-layer information and inter-layer information are combined based on layer relevance. In our previous work, we constructed a multilayer protein network and applied it for the detection of protein complexes (Li et al., 2016) and for the prediction of protein functions (Zhao et al., 2016a). In this study, we propose a tensorial framework to represent the newly constructed multiplex biological network, and we aim to apply it for the identification of essential proteins by extending the random walk with restart algorithm. Our experimental results demonstrated that our proposed MON approach outperformed six types of centrality approaches, including DC (Hahn and Kern, 2004), IC (Stephenson and Zelen, 1989), CC (Wuchty and Stadler, 2003), BC (Joy et al., 2005), SC (Estrada and Rodriguez-Velazquez, 2005), and NC (Wang et al., 2011) and five types of network topological features and biological data sources fusion-based approaches such as PeC (Li et al., 2012), CoEWC (Zhang et al., 2013), POEM (Zhao et al., 2014), ION (Peng et al., 2012), and FDP (Zhang F. et al., 2019).

## Materials

To estimate the performance of MON, we used it to identify essential proteins in the PIN of *Saccharomyces cerevisiae* that was derived from the database of interacting proteins (DIP) (Xenarios et al., 2002) and Gavin datasets (Gavin et al., 2006). The PINs from *Saccharomyces cerevisiae*, which have been well-characterized by a number of studies, are the most complete and comprehensive. After removing self-interactions and repeated interactions, the DIP dataset finally obtained 5,093 proteins and 24,743 interactions, and the Gavin dataset consisted of 1,855 proteins and 7,669 interactions. The domain data for building the multiplex biological network was downloaded from the Pfam database (Punta et al., 2011). The gene expression profile (Tu et al., 2005) of the yeast was derived from GSE3431 in the GEO (Gene Expression Omnibus) that contained the expression values of 6,776 genes at 36 moments, where 4,985 and 1,827 of these genes were located in the DIP and Gavin PINs, respectively. The gene coverage rates of the two PINs in gene expression profile were all >95% (DIP: 4,985/5,093 = 97.88%, Gavin: 1,827/1,855 = 98.49%). Information on orthologous proteins was obtained from the InParanoid database (Östlund et al., 2009) (Version 7) that consisted of a collection of pairwise comparisons between 100 whole genomes. A benchmark set of essential proteins from *Saccharomyces cerevisiae* that consisted of 1,285 essential proteins was derived from the MIPS (MIPS: analysis and annotation of proteins from whole genomes in 2005) (Mewes et al., 2006), saccharomyces genome database (SGD) (Cherry et al., 2011), and database of essential genes (DEG) (Zhang and Lin, 2008) databases. Among the 5,093 proteins in the DIP network, 1,167 proteins were essential and 3,526 proteins were non-essential. In the Gavin dataset, the number of essential proteins and non-essential proteins was 714 and 1,141, respectively. Table 1 lists the details of the two yeast PINs.

**Table 1:** Details of two yeast protein interaction networks.

**Dataset**	**Proteins**	**Interactions**	**Essential proteins**	**Expressed proteins**
DIP	5,093	24,753	1,167	4,985
Gavin	1,855	7,669	714	1,927

## Methods

**Figure 1:**
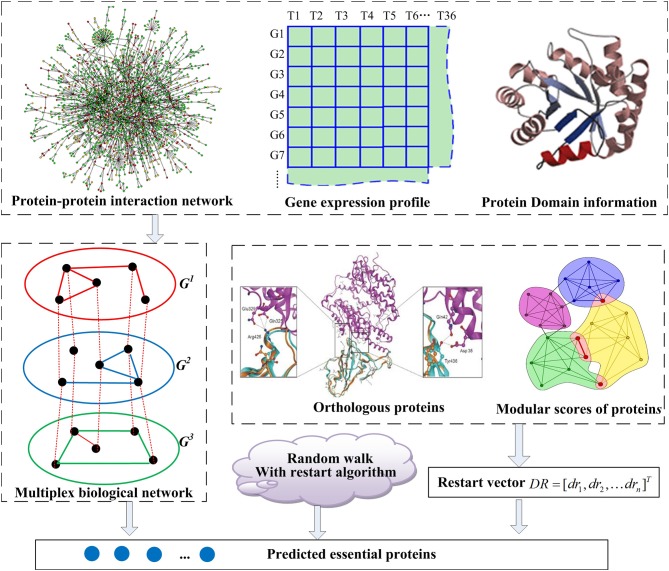
The flowchart of MON method. A multiplex biological network *G* = (*G*^1^, *G*^2^, *G*^3^) is constructed with integration of protein interaction networks (PINs), gene expression profile, and protein domain information, firstly. And then, a restart vector is established according to orthologous proteins and module scores of proteins. Based on these, the random walk with restart algorithm is applied to score and rank essential proteins.

The outline for the entire MON approach includes (1) establishing a multiplex biological network by integrating the topology of PINs, protein domains, and gene expression profile, (2) extending the random walk with restart algorithm to the tensor model corresponding to the multiplex biological network, and (3) sorting proteins in descending order, with the top *K* of these proteins being exported. The flowchart for the MON approach is provided in Figure 1.

### Construction of Multiplex Biological Networks

For our purpose, we consider a multiplex biological network *G* = (*G*^1^, *G*^2^,…, *G*^*L*^), where *G*^*i*^ = (*V, E*^*i*^) represents the network of the layer of *i*. *V* = {*v*_1_, *v*_2_,…, *v*_*n*_} is a set of sharing proteins for all layers in *G*, and *E*^*i*^ = {*e*_*i*__1_, *e*_*i*__2_,…, *e*_*im*_} is a set of interactions at *i*-th layer in the multiplex biological network *G*.

In this study, we constructed a multiplex biological network *G* = (*G*^1^, *G*^2^, *G*^3^) by integrating PINs, gene expression profiles, and protein domain information. In the first layer, a co-neighbor network (CN) was established through the analysis of the topology characteristics of PINs, while in the second layer, a co-structure network was constructed according to the correlation analysis based on the protein domain information. In the third layer, a co-expression network was related to the property of co-expression derived from time course gene expression profiles.

#### Co-neighbor Network *G*^1^

The CN was established by exploring common neighbors between pairs of proteins. Intuitively, the greater number of common neighbors that the two proteins possess, the more credible the interactions between these two proteins will be. If two proteins *p*_*i*_ and *p*_*j*_ interact with each other in PINs and share at least one common neighbor, they will connect to each other within the CN. The weight of interaction between *p*_*i*_ and *p*_*j*_ can be calculated by the following formula:

(1) $$\begin{array}{l} e^{1}\left(i,j\right)=\{\begin{array}{cc} \frac{|N_{i}⋂N_{j}{|}^{2}}{\left(|N_{i}|-1\right)\times \left(|N_{j}|-1\right)}, & if|N_{i}⋂N_{j}|>\text{0}\\ 0 & ,\;otherwise \end{array} \end{array}$$

where *N*_*i*_ and *N*_*j*_ represent the direct neighbors set of *p*_*i*_ and *p*_*j*_, respectively, and *N*_*i*_ ∩ *N*_*j*_ denotes the common neighbors set for protein *p*_*i*_ and protein *p*_*j*_.

#### Co-structure Network *G*^2^

Domains are sequential and structural motifs that are found independently in different proteins and act as the stable functional blocks of proteins. Based on this, we created the co-structure network based on data from protein domains. First, we analyzed the importance of proteins relative to the domains based on the association between proteins and domains. Given a protein *p*_*i*_, its domain score *P*_*D* can be calculated as follows:

(2) $$\begin{array}{l} P\text{\_}D\left(P_{i}\right)=\overset{\left|D\right|}{\underset{j=1}{\sum }}\frac{1}{NP_{j}}\times t_{ij} \end{array}$$

In Equation (2), *D* is a list of distinct categories of domains related to all proteins. *NP*_*j*_ is the number of proteins that contain the domain *d*_*j*_. If the protein *p*_*i*_ contains the domain *d*_*j*_, *t*_*ij*_ is assigned the value of 1. Otherwise, *t*_*ij*_ is set to 0. Finally, the *P*_*D* score of *p*_*i*_ can be normalized and calculated as follows:

(3) $$\begin{array}{l} P\text{\_}D\left(p_{i}\right)=\frac{P\text{\_}D\left(p_{i}\right)-\underset{1\leq j\leq |P|}{\min}\left(P\text{\_}D\left(p_{j}\right)\right)}{\underset{1\leq j\leq |P|}{\max}\left(P\text{\_}D\left(p_{j}\right)\right)-\underset{1\leq j\leq |P|}{\min}\left(P\text{\_}D\left(p_{j}\right)\right)} \end{array}$$

From the above equation, we can easily determine that the value of *P*_*D* falls into the interval [0, 1]. From this perspective, the *P*_*D* score of a protein can be interpreted as its probability of becoming an essential protein. Moreover, previous studies (Stephenson and Zelen, 1989) have indicated that essential genes or proteins tend to form essential modules through their interactions. We assumed that the essential probabilities of proteins mentioned above were independent of each other. The probability (or weight) of interaction between two proteins *p*_*i*_ and *p*_*j*_ in the co-structure network can be calculated as follows.

(4) $$\begin{array}{l} e^{2}\left(i,j\right)=P\text{\_}D\left(p_{i}\right)\times P\text{\_}D\left(p_{j}\right) \end{array}$$

#### Co-expression Network *G*^3^

The Pearson's correlation coefficient (PCC) was adopted to evaluate the co-expression probability of a pair of proteins based on gene expression profiles. Let *g*(*p*_*i*_, *j*) denote the expression value of the gene *p*_*i*_ at the *j*-th time point, and then for a pair of genes *p*_*i*_ and *p*_*j*_, the correlation between them can be calculated as follows:

(5) $$\begin{array}{l} PCC\left(p_{i},p_{j}\right)=\frac{n\sum g\left(p_{i},k\right)g\left(p_{j},k\right)-\sum g\left(p_{i},k\right)\sum g\left(p_{j},k\right)}{\sqrt{n\sum g{\left(p_{i},k\right)}^{2}-{\left(\sum g\left(p_{i},k\right)\right)}^{2}}\sqrt{n\sum g{\left(p_{j},k\right)}^{2}-{\left(\sum g\left(p_{j},k\right)\right)}^{2}}} \end{array}$$

Two proteins were regarded as co-expressed if they interacted with each other in the original PINs and their correlation coefficient was not zero. The weight of interaction between *p*_*i*_ and *p*_*j*_ in the co-expression network was set to the absolute value of their correlation coefficient. Specifically, *e*^3^ (*i, j*)= |*PCC* (*p*_*i*_, *p*_*j*_)|.

### Random Walk With Restart on Multiplex Biological Networks

To study the multiplex network systematically, it is necessary to develop a precise mathematical model and appropriate tools. In this paper, we represent the newly constructed multiplex biological network *G* using the tensor model and extend the random walk with restart algorithm.

Let $T=\left(t_{ijk}\right)\in {\mathbb{R}}^{n\times n\times m}$ denote the three-order adjacency tensor corresponding to the multiplex biological network *G* = (*G*^1^, *G*^2^, *G*^3^), where *n* and *m* are the number of proteins and categories of interactions between proteins, respectively. Each element of *T* is defined as follows:

(6) $$\begin{array}{l} t_{ijk}=\{\begin{array}{ccc} e^{k}\left(i,j\right) & , & if\left(p_{i},p_{j}\right)\in E^{k}\\ 0 & , & otherwise \end{array} \end{array}$$

Here 1 ≤ *i, j* ≤ *n*, 1 ≤ *k* ≤ *m* (*m* = 3) and *e*^*k*^ (*i*, j) represents the weight of interaction between *p*_*i*_ and *p*_*j*_ at the *k*-th layer. We can thus extend the random walk with restart algorithm from a two-dimensional matrix to the tensor for scoring proteins. Studies show that the structural characteristics of different layers in multiplex networks are indeed correlated to each other (Jalili et al., 2017). Based on this, we propose that considering the importance of different types of interactions can enhance the performance for the discovery of essential proteins. Our statistics revealed mutually reinforcing relationships between important or key nodes with different types of links pointed to them in multiplex biological networks. Let the vectors $x={\left[x_{1},x_{2},\ldots ,x_{n}\right]}^{T}\in {\mathbb{R}}^{n}$ and $y={\left[y_{1},y_{2},\ldots ,y_{n}\right]}^{T}\in {\mathbb{R}}^{n}$ denote important scores of proteins and different categories of interactions between proteins, respectively. We formally described the relationships between *x* and *y* based on the tensor *T* using the following equation:

(7) $$\begin{array}{l} x=f\left(T,x,y\right),y=g\left(T,x\right) \end{array}$$

The most critical task for us was to design reasonable functions *f* and *g* and to calculate *y* and *z*, respectively. We now propose the idea to define a higher-order Markov chain by normalizing the tensor. This leads to two probability transition tensors $T^{\left(1\right)}=\left({t^{\left(1\right)}}_{ijk}\right)\in {\mathbb{R}}^{n\times n\times l}$ and $T^{\left(2\right)}=\left({t^{\left(2\right)}}_{ijk}\right)\in {\mathbb{R}}^{n\times n\times l}$ that are calculated as follows:

(8) $$\begin{array}{l} t_{i,j,k}^{\left(1\right)}=\{\begin{array}{c} \frac{t_{i,j,k}}{\overset{n}{\underset{i=1}{\sum }}t_{i,j,k}}\;if\overset{n}{\underset{i=1}{\sum }}t_{i,j,k}>0\\ 1/n\;otherwise\; \end{array} \end{array}$$

(9) $$\begin{array}{l} t_{i,j,k}^{\left(2\right)}=\{\begin{array}{c} \frac{t_{i,j,k}}{\overset{m}{\underset{k=1}{\sum }}t_{i,j,k}}\;if\overset{m}{\underset{k=1}{\sum }}t_{i,j,k}>0\\ 1/m\;otherwise \end{array} \end{array}$$

We can then easily obtain the following formulas:

(10) $$\begin{array}{l} 0\leq t_{i,j,k}^{\left(1\right)}\;\leq \text{1,}\overset{n}{\underset{i=1}{\sum }}t_{i,j,k}^{\left(1\right)}=1 \end{array}$$

(11) $$\begin{array}{l} 0\leq t_{i,j,k}^{\left(2\right)}\;\leq \text{1,}\overset{m}{\underset{k=1}{\sum }}t_{i,j,k}^{\left(2\right)}=1 \end{array}$$

Equations (8) and (9) can be interpreted as the transition probabilities of two third-order Markov chains (*X*_*t*_)_*t*∈ℕ_ and (*Y*_*t*_)_*t*∈ℕ_, respectively.

(12) $$\begin{array}{l} t_{i,j,k}^{\left(1\right)}=P\left[X_{t}=i|X_{t-1}=j,Y_{t}=k\right] \end{array}$$

(13) $$\begin{array}{l} t_{i,j,k}^{\left(2\right)}=P\left[Y_{t}=k|X_{t}=i,X_{t-1}=j\right] \end{array}$$

If the last state was the *i*-th node, then the next state is the *j*-th node through the *k*-th type of interaction with probability $t_{i,j,k}^{\left(1\right)}$. Similarly, $t_{i,j,k}^{\left(2\right)}$ can be considered as the probability of selecting the *k*-th type of interaction from the *j*-th node to the *i*-th node. For the calculation of the random variables *X* and *Y*, the above two equations are deduced according to the total probability formula as follows:

(14) $$\begin{array}{l} P\left[X_{t}=i\right]=\overset{n}{\underset{j=1}{\sum }}\overset{m}{\underset{k=1}{\sum }}t_{i,j,k}^{\left(1\right)}\times P\left[X_{t-1}=j,Y_{t}=k\right] \end{array}$$

(15) $$\begin{array}{l} P\left[Y_{t}=k\right]=\overset{n}{\underset{i=1}{\sum }}\overset{n}{\underset{j=1}{\sum }}t_{i,j,k}^{\left(2\right)}\times P\left[X_{t}=i,X_{t-1}=j\right] \end{array}$$

*P*[*X*_*t*−1_ = *j, Y*_*t*_ = *k*] represents the joint probability distribution of *X*_*t*−1_ and *Y*_*t*_, and *P*[*X*_*t*_ = *i, X*_*t*−1_ = *j*] denotes the joint probability distribution of *X*_*t*−1_ and *X*_*t*_. Considering the steady state of the Markov chain, we can obtain the following formulas:

(16) $$\begin{array}{l} x_{i}=\underset{t\to \infty }{\lim}P\left[X_{t}=i\right],\;\left(1\leq i\leq n\right) \end{array}$$

(17) $$\begin{array}{l} y_{k}=\underset{t\to \infty }{\lim}P\left[Y_{t}=k\right],\;\left(1\leq k\leq m\right) \end{array}$$

It is very difficult to calculate *X* and *Y* due to their coupling to each other and the observation that they contain two joint probability distributions in Equations (14) and (15). In this study, we assumed that the random variables *X* and *Y* were completely independent of each other. Thereafter, we could obtain these following formulas:

(18) $$\begin{array}{l} P\left[X_{t-1}=j,Y_{t}=k\right]\text{=}P\left[X_{t-1}=j\right]P\left[Y_{t}=k\right] \end{array}$$

(19) $$\begin{array}{l} P\left[X_{t}=i,X_{t-1}=j\right]\text{=}P\left[X_{t}=i\right]P\left[X_{t-1}=j\right] \end{array}$$

Based on the above assumption and the fact that *t* continues to infinity, Equations (16) and (17) could be deduced as:

(20) $$\begin{array}{l} x_{i}=\overset{n}{\underset{j=1}{\sum }}\overset{m}{\underset{k=1}{\sum }}t_{i,j,k}^{\left(1\right)}x_{j}y_{k},\;i=1,2,\ldots n \end{array}$$

(21) $$\begin{array}{l} y_{k}=\overset{n}{\underset{i=1}{\sum }}\overset{n}{\underset{j=1}{\sum }}t_{i,j,k}^{\left(2\right)}x_{i}x_{j},\;k=1,2,\ldots m \end{array}$$

Based on this, we designed the proper solutions for the functions *f* and *g*. Therefore, the random walk with restart algorithm in the multiplex biological network case could be described as follows:

(22) $$\begin{array}{l} X_{t}=\alpha \times T^{\left(1\right)}\times X_{t-1}Y_{t-1}+\left(1-\alpha \right)\times RV \end{array}$$

(23) $$\begin{array}{l} Y_{t}=T^{\left(\text{2}\right)}\times {X_{t}}^{\text{2}} \end{array}$$

The restart vector, *RV*, represents the initial probability distribution. α is the restart probability. The overall framework of random walk with restart on multiplex biological networks can be illustrated by Algorithm 1.

**Algorithm 1:** Random walk with restart in multiplex biological networks

Input: A multiplex biological network *G*; Restart vector *RV*; Stopping threshold ∂
Output: A vector representing the score of nodes *X*
Step 1. Construct two transition probability tensors *T*^(1)^ and *T*^(2)^ using Equations (8) and (9)
Step 2. Initialize *X*_0_ = 1/*n, Y*_0_ = 1/*m*
Step 3. Let *t* = 1
Step 4. Calculate *X_*t*_* = α × *T*^(1)^ × *X_*t*_*_−1_ × *Y_*t*_*_−1_ + (1-α) × *RV*
Step 5. Calculate *Y_*t*_* = *T*^(2)^ × *X_*t*_*^2^
Step 6. If ||*X_*t*_*- *X_*t*_*_−1_|| + ||*Y_*t*_*-*Y_*t*_*_−1_|| < ∂, then let *X* = *X_*t*_, Y* = *Y_*t*_* and terminate the algorithm. Otherwise, let *t* = *t* + 1, and then go to Step 4.
Step 7. Output *X*

### Identification of Essential Proteins

Thus far, the framework for assessing the importance of proteins in multiplex biological networks has been established. Now, we describe the MON approach that was designed for the identification of essential proteins from multiplex biological networks. Algorithm 2 details the MON approach.

**Algorithm 2:** MON

**Input:** A PIN network, protein domain, gene expression, ortholog data sets, module scores of proteins, and parameter *K*
**Output:** Top *K* proteins sorted by *pr* in descending order
Step 1. Construct a multiplex biological network *G* according to Equations (1)–(5)
Step 2. Calculate initial vector *DR*
Step 3. *pr* = Algorithm1(*G, dr*, ϵ)
Step 4. Sort proteins by the value of *pr* in descending order
Step 5. Output top *K* of sorted proteins

Based on a user-specified output number of top-ranking proteins, *K*, our approach first constructed the multiplex biological network *G* by integrating PINs, gene expression, and protein domains. Then, considering the conservative and modular features of proteins, a vector $DR={\left[dr_{1},dr_{2},\ldots dr_{n}\right]}^{T}$ was initialized using the follow equation:

(24) $$\begin{array}{l} dr\left(p_{i}\right)=\beta \times C\text{\_}S\left(p_{i}\right)+\left(1-\beta \right)\times M\text{\_}S\left(p_{i}\right) \end{array}$$

In the above equation, *C*_*S*(*p*_*i*_) and *M*_*S*(*p*_*i*_) represent conservative score and modular score of the protein *p*_*i*_, respectively. Conservative score of the protein *p*_*i*_ is derived from information from orthologous proteins and is defined as follows (Zhao et al., 2016b):

(25) $$\begin{array}{l} C\text{\_}S\left(p_{i}\right)=\frac{N\left(p_{i}\right)}{\underset{1\leq j\leq |V|}{\max}\left(N\left(p_{j}\right)\right)} \end{array}$$

where *N*(*p*_*i*_) denotes the number of homologous proteins that *p*_*i*_ contains in reference organisms. The modular scores of proteins are output scores of the POEM approach with normalization processing (Zhao et al., 2014). Next, we applied the random walk with restart algorithm to the multiplex biological network *G* and generated a score vector *pr*. Finally, proteins were sorted in descending order according to *pr*, with the top *K* of them being exported.

## Results and Discussion

To evaluate the essential nature of proteins in PINs, they were ranked in descending order based on their ranking scores that were computed by our MON model and by the 11 other competing essential protein prediction approaches, which included DC (Hahn and Kern, 2004), IC (Stephenson and Zelen, 1989), CC (Wuchty and Stadler, 2003), BC (Joy et al., 2005), SC (Estrada and Rodriguez-Velazquez, 2005), NC (Wang et al., 2011), PeC (Li et al., 2012), CoEWC (Zhang et al., 2013), POEM (Zhao et al., 2014), ION (Peng et al., 2012), and FDP (Zhang F. et al., 2019). After this, the top 100, 200, 300, 400, 500, and 600 ranked proteins were selected as candidates for verification as essential proteins. According to the set of known essential proteins, the number of true essential proteins was determined to assess the performance of each approach. Here, we represent the results for the DIP dataset, in detail, and those for the Gavin dataset, in brief.

### Effects of Parameters α and β

**Figure 2:**
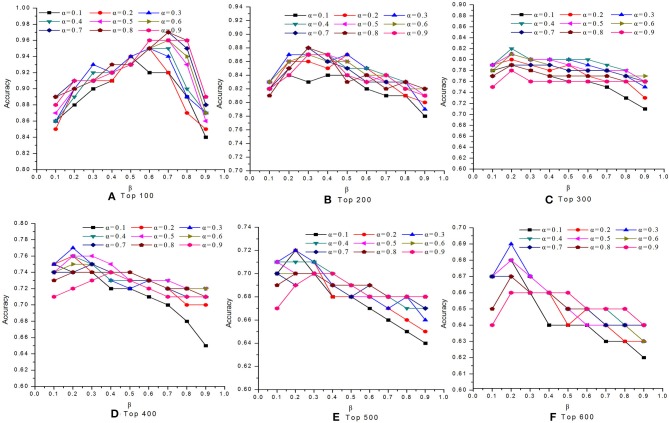
The analysis of parameters α and β on DIP dataset. The figure shows the effect of parameter α and β on the performance of MON on DIP dataset. Six panels represent prediction accuracy of MON in each top percentage of ranked proteins by setting different values of α and β, ranging from 0 to 1. **(A)** Top 100, **(B)** Top 200, **(C)** Top 300, **(D)** Top 400, **(E)** Top 500, **(F)** Top 600.

**Figure 3:**
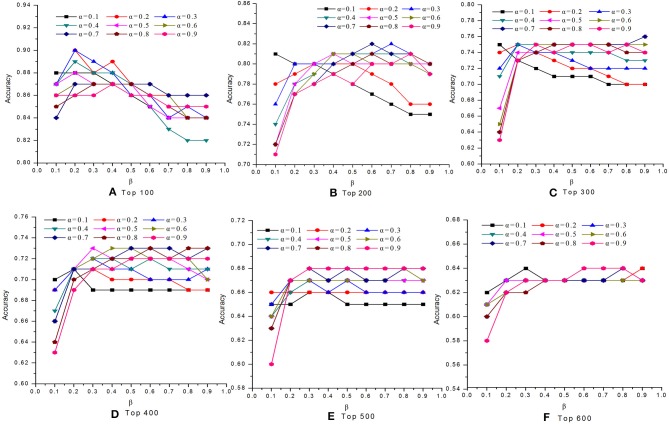
The analysis of parameters α and β on Gavin dataset. The figure shows the effect of parameter α and β on the performance of MON on Gavin dataset. The optimum of α and β for Gavin dataset is 0.3 and 0.2, respectively. **(A)** Top 100, **(B)** Top 200, **(C)** Top 300, **(D)** Top 400, **(E)** Top 500, **(F)** Top 600.

In this study, we introduced two self-defined parameters as α and β. The parameter α (0 < α <1) was used to control the weight of two scores at step 4 of Algorithm 1. The parameter β (0 < β <1) was adopted to adjust the contribution of conservative scores and modular scores of proteins in Equation (24). To study the effects of parameters α and β on the performance of our MON approach, we evaluated the identification accuracy by setting different values for α and β. Figures 2, 3 reveal the comparative results in the DIP and Gavin datasets when the parameters α and β possessed different values between 0 and 1, respectively. We selected top 100, top 200, top 300, top 400, top 500, and top 600 candidate proteins as detected by MON, respectively. The identification accuracy was evaluated by the percentage of true essential proteins in the top candidates. Figure 2 indicates that MON achieves the highest prediction accuracy when α is 0.3 and β is 0.5. Figure 3 shows that the optimum values for α and β for the Gavin dataset are 0.3 and 0.2, respectively.

### Comparison With 11 Other Approaches

**Figure 4:**
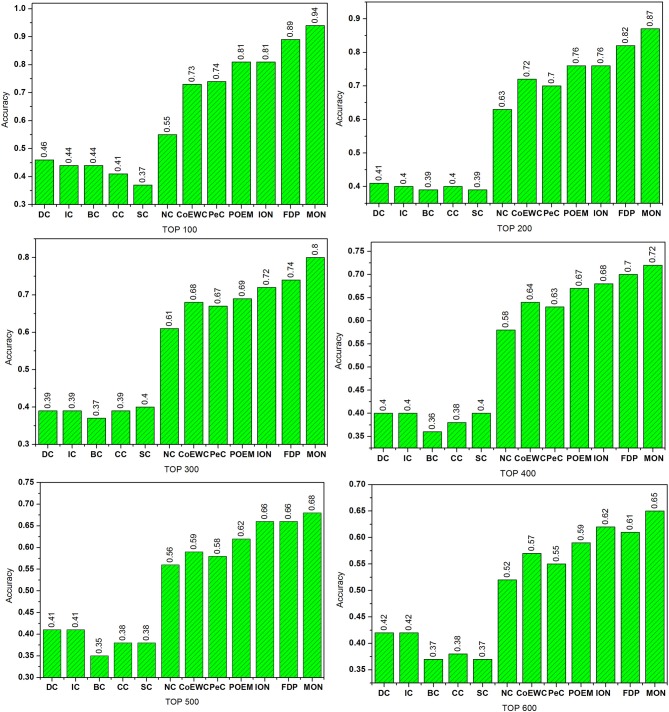
Comparison of the percentage of essential proteins detected by MON and 11 other previously proposed methods. The proteins in protein–protein interaction (PPI) network are ranked in the descending order based on their ranking scores computed by MON, Degree Centrality (DC), Information Centrality (IC), Closeness Centrality (CC), Betweenness Centrality (BC), Subgraph Centrality (SC), Neighbor Centrality (NC), PeC, CoEWC, POEM, ION, and FDP. Then, top 100, 200, 300, 400, 500, and 600 of the ranked proteins are selected as candidates for essential proteins. According to the list of known essential proteins, the percentage of true essential proteins is used to judge the performance of each method. The figure shows the percentage of true essential proteins predicted by each method in each top percentage of ranked proteins. The digits on bars denote the percentage of proteins predicted by each method.

To validate the performance of our MON approach, we made comprehensive comparisons of MON to the 11 other competing essential protein identification approaches. Proteins were ranked in descending order according to their scores obtained from each approach. Several of the top predicted proteins were viewed as essential proteins. Then, by comparing to the benchmark set, we determined how many of these candidate proteins were true essential proteins. Figure 4 reveals the percentage of essential proteins detected by MON and the 11 other prediction approaches within the yeast PIN.

As shown in Figure 4, it is clear that MON allows for a higher predictive performance than that of the other competitive centrality methods. For the top 100 candidate proteins and the top 200 candidate proteins, the prediction accuracy of the MON approach was >86%. MON exhibited improvements of 70.91, 38.10, 31.87, 25.65, 21.51, and 26.45% compared to the values achieved by NC, which possessed the highest prediction accuracy among the six network topology-based centrality methods (DC, IC, BC, CC, SC, and NC) when selecting from the top 100 to top 600 proteins. In particular, when selecting the top 200 proteins, the accuracy of MON in predicting essential proteins was still close to 90%, and this was higher than that of DC, IC, BC, CC, SC, NC, CoEWC, PeC, POEM, and ION for predicting the top 100 proteins. Compared to FDP, which obtained the best prediction accuracy of all 11 competitive approaches, the performance of MON was improved by 5.62, 6.10, 7.62, 3.21, 2.73, and 6.52% from the top 100 to top 600 proteins, respectively.

### Validated by Precision-Recall Curves

**Figure 5:**
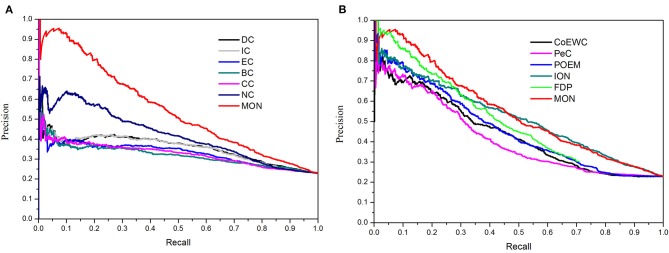
Precision-recall (PR) curves of MON and 11 other existing centrality methods. The proteins ranked in top K (cutoff value) by each method (MON, DC, IC, SC, BC, CC, NC, PeC, CoEWC, POEM, ION, and FDP) are selected as candidate essential proteins (positive data set), and the remaining proteins in PPI network are regarded as candidate non-essential proteins (negative data set). With different values of K selected, the values of precision and recall are computed for each method. The values of precision and recall are plotted in PR curves with different cutoff values. **(A)** Shows the PR curves of MON, DC, IC, SC, BC, CC, and NC. **(B)** Shows the PR curves of MON and other five methods: PeC, CoEWC, POEM, ION, and FDP.

Additionally, the precision-recall (PR) curve was adopted to evaluate the overall performance of MON and the other 11 approaches. First, the proteins in PINs were ranked in a descending order based on the scores obtained from each approach. Next, the top *K* proteins were selected and placed into the positive set (candidate essential proteins), while the rest of the proteins were stored in the negative set (candidate non-essential proteins). The cutoff parameter of *K* ranged from 1 to 5,093. Based on different selected values of *K*, the values of precision and recall were calculated by each approach. Finally, the PR curves were plotted according to values of precision and recall when *K* changed from 1 to 5,093. Figure 5A shows the PR curves of MON and six topology-based centrality methods (DC, IC, BC, CC, SC, and NC). Figure 5B illustrates the PR curves for MON and the other five approaches (PeC, CoEWC, POEM, ION, and FDP). Figure 5 indicates that the PR of MON is clearly higher than that of all competing approaches.

### Validated by Jackknife Methodology

**Figure 6:**
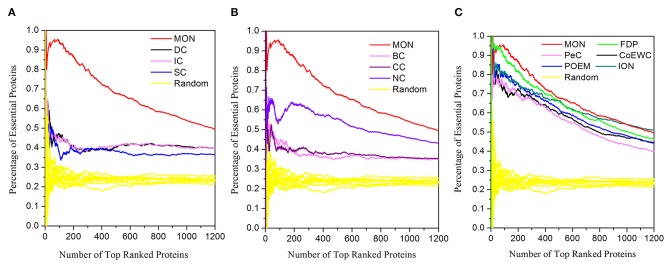
Jackknife curves of the 12 methods. The x-axis represents the proteins in protein–protein interaction (PPI) network ranked by MON and 11 other methods, ranked from left to right as strongest to weakest identification of essentiality. The Y-axis is the percentage of essential proteins encountered moving left to right through the ranked. The areas under the curve for MON and the 11 other methods are used to compare their prediction performance. In addition, the 10 random assortments are also plotted for comparison. **(A)** Shows the comparison results of MON, DC, IC, SC, and DC. **(B)** Represents the comparison results of MON, BC, CC, and NC. **(C)** Illustrates the comparison results of MON and other five methods: PeC, CoEWC, POEM, ION, and FDP.

A further comparison between the novel approach MON and the 11 other competing approaches (DC, BC, CC, SC, IC, NC, UDoNC, PeC, CoEWC, POEM, ION, and FDP) was performed by adopting the jackknife methodology (Holman et al., 2009). The areas under the jackknife curve for each approach were used to evaluate their accuracy in identifying essential proteins. Additionally, 10 random assortments were also depicted for this comparison. Figure 6 illustrates the comparison results where the horizontal axis represents the proteins ranked in descending order according to their scores calculated by each approach and the vertical axis is the percentage of essential proteins related to ranked proteins. Figure 6A shows the comparison results between MON and three topology-based centrality methods (DC, IC, and SC). Figure 6B represents the comparison results between MON and three centrality methods (BC, CC, and NC). Figure 6C indicates the comparison results between MON and the remaining five approaches (PeC, CoEWC, POEM, ION, and FDP). As shown in Figure 6, it is clear that the jackknife curve for MON is evidently better than that of the 11 previously proposed approaches. Moreover, MON and the 11 other competing approaches had all achieved improved identification performance compared to that of randomized sorting.

### Analysis of the Differences Between MON and Other Approaches

To analyze why and how MON obtains high performance for the identification of essential proteins, we investigated the relationship and differences between MON and the 11 other competitive approaches by detecting a small fraction of proteins. For each approach, the top 100 proteins were selected and compared. The number of top 100 identified proteins ranked by each approach is listed in Table 2.

**Table 2:** Common and different proteins predicted by MON and other competing methods ranked in top 100 proteins.

**Methods (Mi)**	**|MON∩Mi|**	**Non-essential proteins in {Mi – MON}**	**Percentage of non-essential proteins in {Mi – MON}with low MON (%)**
DC	8	54	88.89
IC	8	56	89.28
SC	8	63	92.06
BC	4	56	87.5
CC	7	59	89.83
NC	25	42	92.96
PeC	56	22	81.82
CoEWC	54	24	83.33
POEM	62	14	92.96
ION	54	19	52.63
FDP	48	8	75

*The table shows the common and the difference between MON and 11 other competing methods (DC, IC, SC, BC, CC, NC, PeC, CoEWC, POEM, ION, and FDP) when predicting top 100 proteins. |MON∩Mi | denotes the number of proteins predicted by both MON and one of the 11 other methods Mi. {Mi – MON} represents the set of proteins detected by Mi while ignored by MON. |Mi – MON| is the number of proteins in set {Mi – MON}. The last column describes the percentages of different non-essential proteins with low MON score (<0.45) in top 100 proteins*.

First, we compared MON to DC, BC, CC, SC, IC, NC, PeC, CoEWC, POEM, ION, and FDP by statistically analyzing the number of proteins that were commonly detected by MON and any of the 11 other competitive approaches. The number of common and different proteins between MON and any of the other competing approaches is shown in Table 2. In Table 2, |MON ⋂ Mi| represents the number of overlapping proteins identified by MON and by a centrality measure Mi. {Mi – MON} denotes the set of proteins predicted by Mi and not by MON, and |Mi–MON| is the number of proteins predicted by Mi and not by MON.

As illustrated in Table 2, among the top 100 proteins, the proportions of overlapping proteins identified by both MON and DC, BC, CC, SC, and IC are all <10%, while the proportions of overlapping proteins detected by both MON and NC and FDP are not more than 50%. The proportion of common proteins predicted by both MON and PeC, CoEWC, POEM, and ION is <65%. Such a small overlap between proteins identified by MON and the 11 other approaches indicates that MON provides a special approach that is different from that of the other approaches. The third column in Table 2 denotes the number of non-essential proteins among different proteins predicted by Mi but not by MON. We further analyzed these non-essential proteins that were identified by the 11 other approaches, and we found that more than 87% of these non-essential genes that were predicted by six network topology-based centrality measures (DC, IC, BC, CC, SC, and NC) possessed very low MON ranking scores (<0.45). Similarly, more than 50% of the non-essential proteins predicted by PeC, CoEWC, POEM, and ION possessed very low MON ranking scores (<0.45).

**Figure 7:**
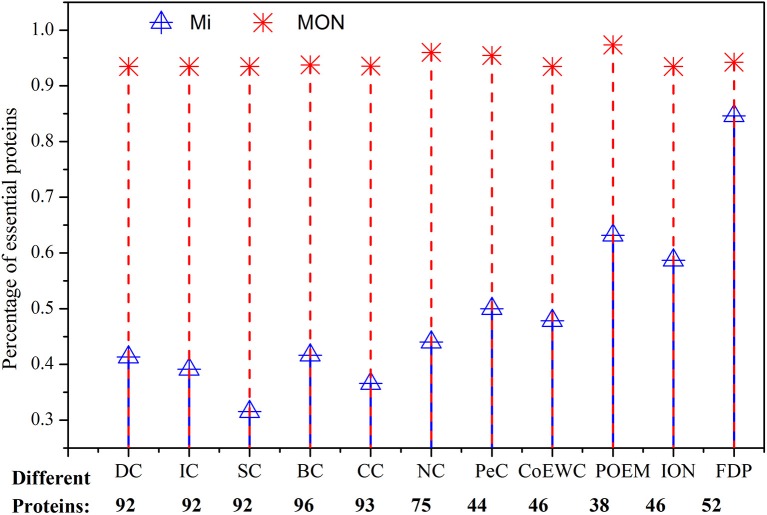
Comparison of the percentage of essential proteins out of all the different proteins between MON and 11 other methods. Different proteins between two prediction methods are the proteins predicted by one method while neglected by the other method. The figure shows how many of the different proteins between MON and 11 other previously proposed methods: DC, IC, SC, BC, CC, NC, PeC, CoEWC, POEM, ION, and FDP are essential. The red dash line represents the percentage of essential proteins detected by MON while ignored by Mi, and the blue solid line denotes the percentage of essential proteins predicted by Mi and not by MON.

Second, we analyzed the essentiality of different proteins detected by MON and by other competing approaches. Figure 7 shows the percentage of essential proteins in all of the various predicted proteins that were detected by MON and the 11 other competitive approaches. In Figure 7, the red dash line represents the percentage of essential proteins detected by MON while ignored by Mi, and blue solid line denotes the percentage of essential proteins predicted by Mi and not by MON. The experimental results shown in Figure 7 illustrate that among these different proteins, the proportion of essential proteins identified by the MON approach is significantly higher than that predicted by the other approaches. In this study, we chose two representative approaches (BC and POEM) as examples to analyze. The former exhibited the largest number of protein differences compared to our MON approach, and the POEM approach possessed the smallest difference compared to the MON approach. Compared to BC, for all of the top 100 predicted proteins, there were 96 different proteins identified by our MON approach. Among these 96 different proteins identified by MON, 93.75% were essential, while only 41.67% proteins predicted by BC were essential. As another example, there were 22 different proteins detected by either MON or by POEM. Among these different proteins, MON could predict more than 95% of the essential proteins, while POEM only discovered <64% of the essential proteins. The comparable results between MON and the other competitive approaches (DC, CC, SC, IC, NC, PeC, CoEWC, and ION) indicate that the proposed MON approach can identify more essential proteins than the other approaches.

Additionally, we selected top 10 identified candidate proteins by our approach as examples to analyze their functional annotations. To this purpose, GO Term (Ashburner et al., 2000) was adopted to characterize these candidate essential proteins, including molecular function (MF), biological process (BP), and cellular component (CC). Table 3 shows the results of functional annotation for these 10 proteins. Out of all the 10 candidate proteins, eight proteins were true essential proteins. And all proteins were annotated in terms of BP, MC, and CC.

**Table 3:** Functional annotations of top 10 predicted essential proteins by MON.

**Proteins**	**Essentiality**	**Go Term**	**Categories**
YDL147W	True	GO:0006511	BP
		GO:0008541, GO:0034515	CC
YFR004W	True	GO:0016579, GO:0043161	BP
		GO:0004843	MF
		GO:0005829, GO:0008541, GO:0034515	CC
YPR108W	True	GO:0006511	BP
		GO:0005198	MF
		GO:0008541	CC
YDL097C	True	GO:0043248, GO:0006511	BP
		GO:0005198	MF
		GO:0008541, GO:0034515	BP
YER012W	True	GO:0010499, GO:0043161	BP
		GO:0005789, GO:0034515	CC
YKL145W	True	GO:0006511, GO:0045899	BP
		GO:0016887	MF
		GO:0008540	CC
YFR052W	True	GO:0006511	BP
		GO:0008541, GO:0034515	CC
YHR200W	False	GO:0006511	BP
		GO:0005198	MF
		GO:0008540	CC
YOR261C	True	GO:0006511	BP
		GO:0008541, GO:0034515	CC
YGR232W	False	GO:0006508	BP
		GO:0005829	CC

*The Table shows results of functional annotation for top 10 proteins predicted by the MON approach. BP, MF, and CC denote biological process, molecular function, and cellular component, respectively*.

### Prediction Performance of MON Based on the Gavin Dataset

**Figure 8:**
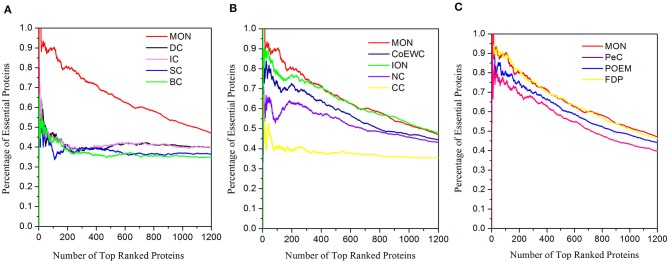
Jackknife curves of MON and 11 other competitive methods based on Gavin dataset. The prediction performance of MON and 11 other existing competitive methods (DC, BC, CC, SC, IC, NC, PeC, CoEWC, POEM, ION, and FDP) based on protein–protein interaction data from Gavin are validated by the jackknife method. **(A)** Shows the Jackknife curves of MON, DC, IC, SC and BC. **(B)** Shows the Jackknife curves of MON, CoEWC, ION, NC and CC. **(C)** Shows the Jackknife curves of MON, PeC, POEM and FDP.

To further test the performance of the proposed approach, we also performed discovery for essential proteins using the Gavin dataset. The ranking scores for proteins were computed using MON (α = 0.3, β = 0.2) and 11 other existing competitive approaches (DC, BC, CC, SC, IC, NC, PeC, CoEWC, POEM, ION, and FDP). The percentage of essential proteins in the top 100, 200, 300, 400, 500, and 600 proteins ranked by these approaches are listed in Table 4. The jackknife curves of each approach are illustrated in Figure 8. All of these experimental results indicate that MON still outperforms the 11 other competitive approaches, using the Gavin dataset. Specifically, when selecting the top 100 ranked proteins, MON resulted in 95.65, 104.55, 143.24, 104.55, 119.51, 63.64, 23.29, 21.62, 11.11, 16.88, and 1.12% improvements compared to the results obtained from DC, IC, CC, BC, SC, NC, PeC, CoEWC, POEM, ION, and FDP, respectively.

**Table 4:** Percentage of essential proteins identified by MON and 11 other competitive methods based on Gavin dataset.

**Methods**	**Top 100 (%)**	**Top 200 (%)**	**Top 300 (%)**	**Top 400 (%)**	**Top 500 (%)**	**Top 600 (%)**
DC	46.00	41.00	38.33	39.50	40.20	41.83
IC	44.00	40.00	39.33	40.25	41.40	41.83
SC	37.00	38.50	39.67	39.50	38.40	36.83
BC	44.00	38.50	37.33	36.25	35.40	36.67
CC	41.00	39.50	39.00	38.25	37.80	38.00
NC	55.00	63.00	60.67	57.50	55.80	51.67
PeC	73.00	72.00	67.67	64.00	59.40	56.83
CoEWC	74.00	69.50	66.67	63.00	58.20	54.67
POEM	81.00	75.50	69.33	66.75	62.00	58.83
ION	77.00	77.00	73.67	70.50	65.80	62.83
FDP	89.00	81.50	75.67	70.25	67.00	63.17
MON	90.00	80.00	74.67	71.25	66.80	62.67

*This table shows the comparison of the percentage of essential proteins predicted by MON and 11 other competitive methods (DC, IC, SC, BC, CC, NC, PeC, CoEWC, POEM, ION, and FDP) based on protein–protein interaction data from Gavin. Since the total number of ranked proteins in Gavin is 1,855*.

## Conclusion

The detection of essential proteins is helpful for understanding the minimum requirements for cell survival and development. Many computational approaches have been proposed that integrate PINs and multi-omics data, and this has led to the identification of multiple interactions or links between proteins. Despite the advances in these approaches, designing efficient algorithms to fuse these multisource biological data remains challenging. A simple strategy is to aggregate a collection of heterogeneous data into a single network; however, this strategy can result in substantial information loss. Studies indicate that different types of biological data sources that possess inherent structural characteristics are correlated to each other. Moreover, high-throughput multi-omics biological data exhibit different degrees of quality and can play various roles in the prediction of essential proteins. The multiplex biological network provides an alternative means to address these problems. In this study, we constructed a multiplex biological network by combining PINs with multi-source biological information, and proposed a new essential proteins prediction approach named MON. In MON, we express the multiplex biological network in the tensor model and extend the random walk with restart algorithm by simulating a higher-order Markov chain. Additionally, the conservative and modular features of essential proteins are both taken into account to improve the performance of MON. The experimental results from two yeast PINs demonstrate that MON performs better than 11 other state-of-the-art approaches for predicting essential proteins.

## Data Availability Statement

Publicly available datasets were analyzed in this study. These data can be found here: https://github.com/husaiccsu/MON.

## Author Contributions

BZ, SH, and LW obtained the protein interaction data, domain data, gene expression profile, and information on orthologous proteins and drafted the manuscript together. BZ and SH designed the new approach, MON, and analyzed the results. XLiu, HX, XH, XLi, and ZZ participated in revising the draft. All authors have read and approved the manuscript.

## Conflict of Interest

The authors declare that the research was conducted in the absence of any commercial or financial relationships that could be construed as a potential conflict of interest.

## References

[B1] Ashburner M. Ball C. A. Blake J. A. Botstein D. Butler H. Cherry J. M.. (2000). Gene ontology: tool for the unification of biology. Nat. Genet. 25, 25–29. 10.1038/75556 10802651 PMC3037419

[B2] Baym M. Shaket L. Anzai I. A. Adesina O. Barstow B. (2016). Rapid construction of a whole-genome transposon insertion collection for *Shewanella oneidensis* by Knockout Sudoku. Nat. Commun. 7:13270. 10.1038/ncomms13270 27830751 PMC5109470

[B3] Cherry J. M. Hong E. L. Amundsen C. Balakrishnan R. Binkley G. Chan E. T.. (2011). Saccharomyces genome database: the genomics resource of budding yeast. Nucl. Acids Res. 40, D700–D705. 10.1093/nar/gkr1029 22110037 PMC3245034

[B4] De Domenico M. Lancichinetti A. Arenas A. Rosvall M. (2015). Identifying modular flows on multilayer networks reveals highly overlapping organization in interconnected systems. Phys. Rev. X 5:011027 10.1103/PhysRevX.5.011027

[B5] Didier G. Brun C. Baudot A. (2015). Identifying communities from multiplex biological networks. PeerJ 3:e1525. 10.7717/peerj.1525 26713261 PMC4690346

[B6] Dubach J. M. Kim E. Yang K. Cuccarese M. Giedt R. J. Meimetis L. G.. (2017). Quantitating drug-target engagement in single cells *in vitro* and *in vivo*. Nat. Chem. Biol. 13:168. 10.1038/nchembio.2248 27918558 PMC5630128

[B7] Estrada E. Rodriguez-Velazquez J. A. (2005). Subgraph centrality in complex networks. Phys. Rev. E 71:056103. 10.1103/PhysRevE.71.056103 16089598

[B8] Gavin A. C. Aloy P. Grandi P. Krause R. Boesche M. Marzioch M.. (2006). Proteome survey reveals modularity of the yeast cell machinery. Nature 440:631. 10.1038/nature04532 16429126

[B9] Hahn M. W. Kern A. D. (2004). Comparative genomics of centrality and essentiality in three eukaryotic protein-interaction networks. Mol. Biol. Evol. 22, 803–806. 10.1093/molbev/msi072 15616139

[B10] Holman A. G. Davis P. J. Foster J. M. Carlow C. K. Kumar S. (2009). Computational prediction of essential genes in an unculturable endosymbiotic bacterium, Wolbachia of *Brugia malayi*. BMC Microbiol. 9:243. 10.1186/1471-2180-9-243 19943957 PMC2794283

[B11] Hsing M. Byler K. G. Cherkasov A. (2008). The use of gene ontology terms for predicting highly-connected 'hub' nodes in protein-protein interaction networks. BMC Syst. Biol. 2:80. 10.1186/1752-0509-2-80 18796161 PMC2553323

[B12] Inouye M. (2016). The first demonstration of RNA interference to inhibit mRNA function. Gene 592, 332–333. 10.1016/j.gene.2016.07.024 27404040

[B13] Jalili M. Orouskhani Y. Asgari M. Alipourfard N. Perc M. (2017). Link prediction in multiplex online social networks. R. Soc. Open Sci. 4:160863. 10.1098/rsos.160863 28386441 PMC5367313

[B14] Joy M. P. Brock A. Ingber D. E. Huang S. (2005). High-betweenness proteins in the yeast protein interaction network. BioMed Res. Int. 2005, 96–103. 10.1155/JBB.2005.96 16046814 PMC1184047

[B15] Lei X. Yang X. Wu F. (2018). “Artificial fish swarm optimization based method to identify essential proteins,” in IEEE/ACM Transactions on Computational Biology and Bioinformatics. 10.1109/TCBB.2018.2865567 30113899

[B16] Li H. Li T. Quang D. Guan Y. (2018). Network propagation predicts drug synergy in cancers. Cancer Res. 78, 5446–5457. 10.1158/0008-5472.CAN-18-0740 30054332

[B17] Li M. Lu Y. Niu Z. Wu F. X. (2015). United complex centrality for identification of essential proteins from PPI networks. IEEE ACM Trans. Comput. Biol. Bioinformatics 14, 370–380. 10.1109/TCBB.2015.2394487 28368815

[B18] Li M. Zhang H. Wang J. X. Pan Y. (2012). A new essential protein discovery method based on the integration of protein-protein interaction and gene expression data. BMC Syst. Biol. 6:15. 10.1186/1752-0509-6-15 22405054 PMC3325894

[B19] Li X. Wang J. Zhao B. Wu F. X. Pan Y. (2016). Identification of protein complexes from multi-relationship protein interaction networks. Hum. Genom. 10:17. 10.1186/s40246-016-0069-z 27461193 PMC4965713

[B20] Mewes H. W. Frishman D. Mayer K. F. Münsterkötter M. Noubibou O. Pagel P.. (2006). MIPS: analysis and annotation of proteins from whole genomes in 2005. Nucl. Acids Res. 34, D169–D172. 10.1093/nar/gkj148 16381839 PMC1347510

[B21] Narasimhan V. M. Hunt K. A. Mason D. Baker C. L. Karczewski K. J. Barnes M. R.. (2016). Health and population effects of rare gene knockouts in adult humans with related parents. Science 352, 474–477. 10.1126/science.aac8624 26940866 PMC4985238

[B22] Östlund G. Schmitt T. Forslund K. Köstler T. Messina D. N. Roopra S.. (2009). InParanoid 7: new algorithms and tools for eukaryotic orthology analysis. Nucl. Acids Res. 38, D196–D203. 10.1093/nar/gkp931 19892828 PMC2808972

[B23] Peng W. Wang J. Cheng Y. Lu Y. Wu F. Pan Y. (2015). UDoNC: an algorithm for identifying essential proteins based on protein domains and protein-protein interaction networks. IEEE ACM Trans. Comput. Biol. Bioinformatics 12, 276–288. 10.1109/TCBB.2014.2338317 26357216

[B24] Peng W. Wang J. Wang W. Liu Q. Wu F. X. Pan Y. (2012). Iteration method for predicting essential proteins based on orthology and protein-protein interaction networks. BMC Syst. Biol. 6:87. 10.1186/1752-0509-6-87 22808943 PMC3472210

[B25] Punta M. Coggill P. C. Eberhardt R. Y. Mistry J. Tate J. Boursnell C.. (2011). The Pfam protein families database. Nucl. Acids Res. 40, D290–D301. 10.1093/nar/gkr1065 22127870 PMC3245129

[B26] Samei Z. Jalili M. (2019). Application of hyperbolic geometry in link prediction of multiplex networks. Sci. Rep. 9, 1–11. 10.1038/s41598-019-49001-7 31471541 PMC6717198

[B27] Stephenson K. Zelen M. (1989). Rethinking centrality: Methods and examples. Soc. Netw. 11, 1–37. 10.1016/0378-8733(89)90016-6

[B28] Tu B. P. Kudlicki A. Rowicka M. McKnight S. L. (2005). Logic of the yeast metabolic cycle: temporal compartmentalization of cellular processes. Science 310, 1152–1158. 10.1126/science.1120499 16254148

[B29] Valdeolivas A. Tichit L. Navarro C. Perrin S. Odelin G. Levy N.. (2019). Random walk with restart on multiplex and heterogeneous biological networks. Bioinformatics 35, 497–505. 10.1093/bioinformatics/bty637 30020411

[B30] Wang J. Li M. Wang H. Pan Y. (2011). Identification of essential proteins based on edge clustering coefficient. IEEE ACM Trans. Comput. Biol. Bioinformatics 9, 1070–1080. 10.1109/TCBB.2011.147 22084147

[B31] Wang P. Gao L. Hu Y. Li F. (2018). Feature related multi-view nonnegative matrix factorization for identifying conserved functional modules in multiple biological networks. BMC Bioinformatics 19:394. 10.1186/s12859-018-2434-5 30373534 PMC6206826

[B32] Wuchty S. Stadler P. F. (2003). Centers of complex networks. J. Theor. Biol. 223, 45–53. 10.1016/S0022-5193(03)00071-7 12782116

[B33] Xenarios I. Salwinski L. Duan X. J. Higney P. Kim S. M. Eisenberg D. (2002). DIP, the Database of Interacting proteins: a research tool for studying cellular networks of protein interactions. Nucl. Acids Res. 30, 303–305. 10.1093/nar/30.1.303 11752321 PMC99070

[B34] Zeng X. Liao Y. Liu Y. Zou Q. (2017). Prediction and validation of disease genes using HeteSim Scores. IEEE ACM Trans. Comput. Biol. Bioinformatics 14, 687–695. 10.1109/TCBB.2016.2520947 26890920

[B35] Zhang F. Peng W. Yang Y. Dai W. Song J. (2019). A novel method for identifying essential genes by fusing dynamic protein–protein interactive networks. Genes 10:31 10.3390/genes10010031 PMC6356314 30626157

[B36] Zhang R. Lin Y. (2008). DEG 5.0, a database of essential genes in both prokaryotes and eukaryotes. Nucl. Acids Res. 37, D455–D458. 10.1093/nar/gkn858 18974178 PMC2686491

[B37] Zhang W. Xu J. Zou X. (2019). Predicting essential proteins by integrating network topology, subcellular localization information, gene expression profile and GO annotation data. IEEE ACM Trans. Comput. Biol. Bioinformatics. 10.1109/TCBB.2019.2916038 31095490

[B38] Zhang X. Xu J. Xiao W. X. (2013). A new method for the discovery of essential proteins. PLoS ONE 8:e58763. 10.1371/journal.pone.0058763 23555595 PMC3605424

[B39] Zhao B. Hu S. Li X. Zhang F. Tian Q. Ni W. (2016a). An efficient method for protein function annotation based on multilayer protein networks. Human Genom. 10:33. 10.1186/s40246-016-0087-x 27678214 PMC5039885

[B40] Zhao B. Wang J. Li M. Wu F. X. Pan Y. (2014). Prediction of essential proteins based on overlapping essential modules. IEEE Trans. nanobioscience, 13, 415–424. 10.1109/TNB.2014.2337912 25122840

[B41] Zhao B. Wang J. Li X. Wu F. X. (2016b). Essential protein discovery based on a combination of modularity and conservatism. Methods, 110, 54–63. 10.1016/j.ymeth.2016.07.005 27402354

